# The 1992 International Symposium on Laboratory Automation and Robotics

**DOI:** 10.1155/S1463924692000385

**Published:** 1992

**Authors:** David J. Curran


					Journal of Automatic Chemistry, Vol. 14, No. 6 (November-December 1992), p. 199
The 1992 International Symposium on
Laboratory Automation and Robotics
1992 was the 10th anniversary of the International Symposium on Laboratory Automation and Robotics. Held
in Boston from 25 to 28 October, the meeting attracted an international audience of425 attendees who came to
hear over 50 oral presentations and view 25 poster papers. As in past years, the format for the conference
included keynote speakers, other oral presentations, poster papers and a visit to the Zymark Corporation
facilities in Hopkinton, Massachusetts. Keynote addresses were presented on Monday morning and were
followed by parallel sessions, one on data management/data handling and one on validating pharmaceutical
laboratory automation. The latter continued into the afternoon along with a session on laboratory workstations.
Poster papers were presented between 3.00 and 5.00 p.m. Tuesday morning began with a session on managing
laboratory automation and was followed by parallel sessions on automating pharmaceutical methods,
environmental, and custom automation solutions, each of which continued on into the afternoon. On
Wednesday morning there were parallel sessions on chemical analysis and biology/biotechnology.
The first keynote lecture was delivered by Eugene J. McGonigle ofthe Schering-Plough Research Institute on:
'Practical aspects of laboratory automation in pharmaceutical development'. Sean O'Connor of Bristol-Myers
Squibb Pharmaceutical Research Institute followed with a keynote address: 'Rational drug design, planned
serendipity and the place of automation in pharmaceutical lead discovery'.
The presentation of the 'Pioneers in Laboratory Robotics' awards took place on Monday morning following
the keynote addresses. The 1992 recipients were:
LeEtta Jarvis (University ofMinnesota, St. Paul)
Julie Tomlinson Glaxo, Inc.)
Kyle Hama (Syntex Discovery Research)
Joseph Franehetti (SmithKline Beecham Pharmaceuticals)
Frank Settle (Virginia Military Institute)
Randy Simpson (United States Department ofAgriculture)
Dieter Fridel (Bayer AG)
Paul Morabito (Dow Chemical)
Ronald Anstey Wyeth-Ayerst Research)
Robert W. Taylor (Los Angeles County Sheriff's Department)
Ko Rick Lung (Du Pont Merck Pharmaceutical Co.).
As automation takes on more of the tasks in the laboratory and as the quality of results, throughput, and
competitive performance become increasingly important, validation becomes more and more significant. This
was a major theme for a number ofpapers at the Symposium. The abstracts ofthe full programme are included
in this issue ofJournal ofAutomatic Chemistry through the co-operation of Zymark Corporation; the papers will be
available in early 1993 as Advances in Laboratory Automation and Robotics, Vol. IX.
This writer highly recommends this meeting, including the visit to Zymark Corporation. The 11th symposium
will be held in Boston from 17 to 20 October 1993. Speaker abstracts are due by 15 March 1993 and poster
abstracts by 15 July 1993.
Further information is availablefrom Christine O'Neil, Zymark Corporation, Zymark Center, Hopkinton, MA 01748, USA
Tel.: 508-435-9500 ext. 2224; fax: 508-435-3439.
David J. Curran
American Editor
199

				

